# Impact of Light-Activated Nanocomposite with Erythrosine B on *agr* Quorum Sensing System in *Staphylococcus aureus*

**DOI:** 10.3390/antibiotics14101010

**Published:** 2025-10-11

**Authors:** Larysa Bugyna, Ľubomír Švantner, Katarína Bilská, Marek Pribus, Helena Bujdáková

**Affiliations:** 1 Department of Microbiology and Virology, Faculty of Natural Sciences, Comenius University in Bratislava, Mlynská dolina, Ilkovičova 6, 842 15 Bratislava, Slovakiasvantner13@uniba.sk (Ľ.Š.); bilska6@uniba.sk (K.B.); 2 Institute of Laboratory Medicine, Faculty of Medicine, Slovak Medical University, Limbova 14, 833 03 Bratislava, Slovakia; larysa.bugyna@szu.sk; 3 Institute of Inorganic Chemistry, Slovak Academy of Sciences, Dúbravská Cesta 9, 845 36 Bratislava, Slovakia; marek.pribus@savba.sk

**Keywords:** *Staphylococcus aureus*, photodynamic inactivation, erythrosine B, nanocomposite, *agr* quorum sensing system

## Abstract

Backround: The *agr* (accessory gene regulator) quorum sensing (QS) system of *Staphylococcus aureus* participates significantly in its virulence and biofilm formation—either through its activation or suppression. The aim of this study was to investigate the impact of photoactive nanomaterials that have been functionalized with erythrosine B (EryB) on the modulation of this *agr* QS system on three methicillin-resistant *S. aureus* (MRSA). Methods: The functionality of the *agr* system was determined by the CAMP test and by quantitative PCR (qPCR) to analyze the expression of the *hld* gene, which is located within the RNAIII and encodes δ-hemolysin. The biofilm was evaluated by crystal violet assay and fluorescence microscopy. The anti-biofilm activity was determined by calculating the colony-forming units. The relative expression of the *hld* gene, determined by qPCR. Results: Using the CAMP test, S66 and S68 strains were found to be *agr*-positive, and strain S73 was *agr*-negative. The relative expression of the *hld* gene increased only in the *agr*-positive strains (600- and 1000-fold). In these strains, the biofilm was less compact compared to the dense biofilm formed by the *agr*-negative strain. The anti-biofilm effectiveness on the nanocomposite with EryB after irradiation reduced the growth of biofilm cells by 100- to 1000-fold compared to the biofilm on polyurethane alone. The qPCR results showed a significant decrease in the relative expression of the *hld* gene in the *agr*-positive strains after irradiation compared to the non-irradiated samples. Conclusions: These results suggest that photoactive nanocomposites with EryB can significantly reduce biofilm formed by MRSA strains, regardless of the functionality of the *agr* QS system.

## 1. Introduction

Methicillin-resistant *S. aureus* (MRSA) [1,2,3,4] has remained a continual problem in both hospital- and community-acquired infections [5,6,7,8]. These resistant bacteria are particularly relevant in connection with the formation of biofilms [9,10,11] on abiotic and biotic surfaces, such as catheters, implants, or damaged tissue in wounds or heart valves and bones [11,12]. The development of *S. aureus* biofilm is a complex process regulated by the quorum sensing (QS) system—a signaling mechanism used for communication within the microbial community [13,14,15].

**Figure 1:**
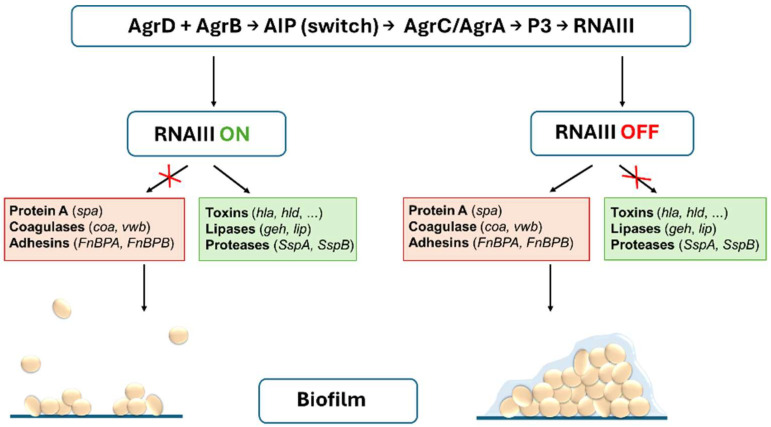
The role of RNAIII in the regulation of virulence factors and biofilm formation in *S. aureus*. RNAIII activated by the *agr* system suppresses the expression of protein A (*spa*), coagulases (*coa, vwb*), adhesins (*fnb*), and biofilm formation, while promoting the expression of toxins (*hla*, *hld*), lipases (*geh*, *lip*), and serine proteases (*ssp*). Suppression of RNAIII leads to inhibition of the release of toxins, lipases, and proteases and the promotion of surface antigens that facilitate the formation of dense biofilm.

The *agr* (accessory gene regulator) QS system [13,14,15,16,17] plays a key role in virulence, biofilm formation, and the adaptation of *S. aureus* to environmental conditions [16,18,19]. Activation of the *agr* system is mediated by an autoinducing peptide (AIP) that accumulates in the extracellular space with increasing bacterial density. AIP binds to the membrane sensor AgrC (sensory histidine kinase), which leads to phosphorylation of the response regulator AgrA. This triggers transcription from the P2 and P3 promoters, which leads to auto-amplification of the agr operon and activates the transcription of RNAIII (a 514-nucleotide-long regulatory RNA). RNAIII is the main effector molecule of the *agr* system that controls more than 200 genes, including those encoding many virulence factors [20,21,22,23,24,25]. RNAIII positively regulates the expression of secreted toxins and enzymes, such as *α*-hemolysin (*hla*) [26,27], *δ*-hemolysin (*hld*) [28,29,30], and serine proteases (*ssp*) [31]. On the other hand, RNAIII inhibits the expression of surface adhesins, such as protein A (*spa*) [32,33], which facilitate adherence [31,34]. Activation of the *agr* system also leads to decreased biofilm production, probably by reducing the amount of the aforementioned surface adhesins and promoting the expression of proteases that contribute to the disruption of biofilm structure and dispersion of biofilm cells [21,35]. The activity of the *agr* system can be assessed using the Christie–Atkins–Munch-Peterson (CAMP) test to evaluate *δ*-toxin production as the main marker of *agr* activity [36,37]. While the *agr*-positive strains are typically associated with the acute phase of infection, the *agr*-negative strains are usually characterized by increased biofilm stability and suppressed δ-toxin production. These strains are generally less virulent in acute infections, but they may be associated with persistent biofilm-related infections [38,39,40]. A simplified model of the effect of the *agr* QS system on virulence factors and biofilm formation is presented in Figure 1.

One of the most promising antimicrobial strategies to combat biofilms is photodynamic inactivation (PDI) [41,42,43,44]. This approach uses a photosensitizer (PS) [45,46], which generates singlet oxygen (^1^O_2_) and reactive oxygen species (ROS), such as hydrogen peroxide (H_2_O_2_) and hydroxyl radicals (-OH), when irradiated with light in the presence of molecular oxygen [47,48,49,50]. These ROS damage cell membranes, proteins, lipids, and nucleic acids, leading to the death of microorganisms [48,49,51]. One of the most promising photosensitizers is erythrosine B (EryB), which has been approved by the Food and Drug Administration (FDA) [52] for use in the pharmaceutical industry [45]. Its absorption maximum is in the green spectral range (~530 nm) [53]. Recent advances have enabled the integration of PS into nanocomposites to enhance the anti-biofilm activity of PDI [53,54,55] against *S. aureus*, showing potential for the modification of medical device surfaces [12,53,54,56].

This study aimed to evaluate the effect of photoactive nanomaterials that have been functionalized with EryB on the modulation of the *agr* QS system, which plays a key role in virulence regulation and is associated with biofilm formation, either through its activation or suppression. This was also the reason why we used clinical MRSA isolates with different *agr* QS system activities for the presented study. Additionally, investigating PDI in relation to the *agr* QS system in the clinically important bacterium *S. aureus* expands knowledge of antimicrobial properties of nanomaterial functionalized with the photoactive molecule EryB.

## 2. Results

### 2.1. Activity of the agr QS System and Biofilm Formation

**Figure 2:**
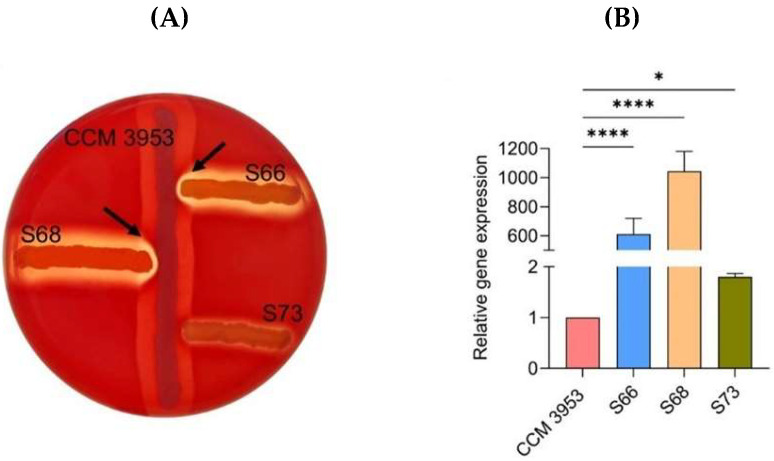
Determination of the functionality of the *agr* QS system in *S. aureus*. (**A**) CAMP test, with arrows indicating expansion of the hemolysis zone as a result of *δ*-hemolysin production; (**B**) relative changes in the *hld* gene expression in planktonic cells of *S. aureus* strains of S66, S68, and S73 compared to standard strain CCM 3953, set to 1; the *rpoD* gene was used as the housekeeping gene. A *p* < 0.05 (*) was considered statistically significant and *p* < 0.0001 (****) extremely significant.

In this study, three clinical isolates of *S. aureus* with different activities of the *agr* QS system and the standard strain CCM 3953 (corresponding to RN4220) were used for experiments. The activity of the *agr* QS system was characterized phenotypically by the modified CAMP test (Figure 2A). Clinical MRSA strains of S66 and S68 showed enhanced hemolysis (Figure 2A, indicated by arrows), indicating the production of *α*- and *δ*-hemolysin, which suggests a functional *agr* system (*agr*-positive). In contrast, strain S73 did not show enhanced hemolysis, indicating a non-functional *agr* system (*agr*-negative). The results observed in the CAMP test were also confirmed by quantitative real-time PCR (qPCR) based on the expression of the *hld* gene, which is located within RNAIII and encodes δ-hemolysin (Figure 2B). This experiment was performed with planktonic cells of *S. aureus*. Strains S66 and S68 showed more than 600- and 1000-fold increase in relative *hld* gene expression, respectively, compared to the standard strain CCM 3953. Strain S73 showed only a 1.8-fold increase in *hld* gene expression compared to CCM 3953, which is not considered relevant from a molecular point of view. These results are consistent with the observed weak hemolytic activity.

**Figure 3:**
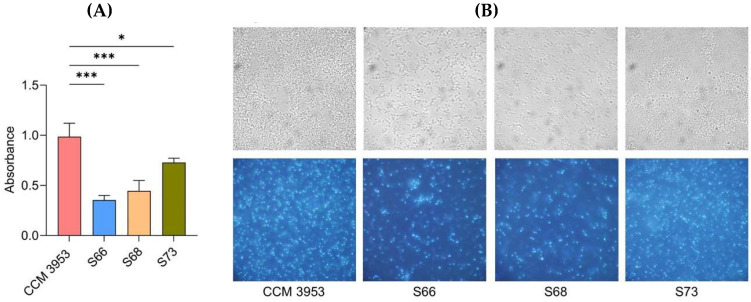
Biofilm formation by *agr*-positive and *agr*-negative strains of *S. aureus*. (**A**) Evaluation of biofilm formation by crystal violet assay; (**B**) (**above**): light microscopy of native 24-h biofilms of tested strains; (**bottom**): fluorescence microscopy of 24-h biofilms formed on slides stained with DAPI. The magnification was 1000×. A *p* < 0.05 (*) was considered statistically significant and *p* < 0.001 (***) highly significant.

The ability to form biofilm was assessed by crystal violet assay (Figure 3A) and supported by microscopic observation (Figure 3B). The results, summarized in Figure 3A, showed that the standard strain CCM 3953 and *agr*-negative MRSA strain S73 were strong biofilm producers, while the *agr*-positive strains S66 and S68 produced less biofilm. Fluorescence microscopy with DAPI staining confirmed dense and compact biofilm layers of strains CCM 3953 and S73. On the other hand, the *agr*-positive strains S66 and S68 formed scattered biofilm clusters with low density (Figure 3B).

### 2.2. Anti-Biofilm Effectiveness of Nanocomposite Functionalized with EryB

**Figure 4:**
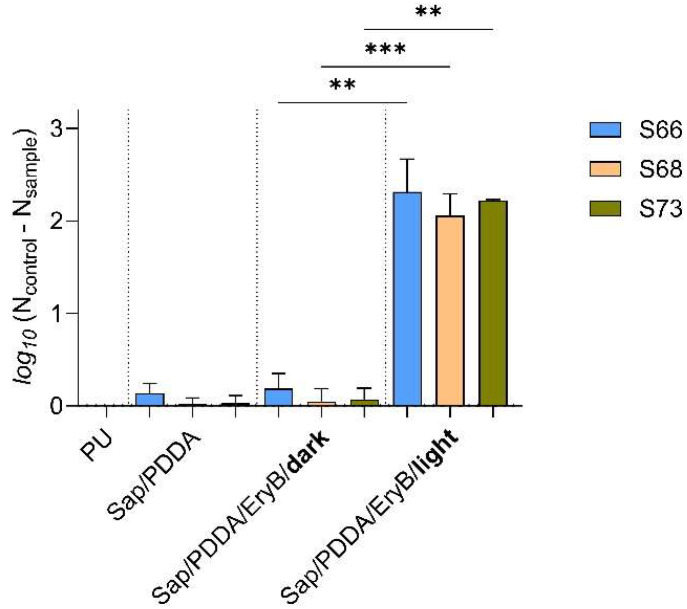
Anti-biofilm effectiveness of nanocomposites against 24-h biofilms of clinical isolates of *S. aureus* S66, S68, and S73 before and after irradiation. The function was *log_10_* (N_control_ − N_sample_); N_control_ represents the number of CFU/mL on control PU material; N_sample_ represents the number of CFU/mL on PU material with modified surface (Sap/PDDA; Sap/PDDA/EryB). Control sample with EryB but cultivated in the dark is labeled **dark**, and sample irradiated with a green laser for 10 min is labeled **light**. A *p* < 0.01 (**) indicates very significant and *p* < 0.001 (***) highly significant.

The nanocomposite disks based on modified polyurethane (PU) were prepared as described by Bugyna et al. [53]. Organoclay dispersion with EryB (Sap/PDDA/EryB), used in modification, was prepared by functionalization of a synthetic layered silicate saponite Sumecton (Sap) and poly (diallyldimethylammonium chloride) (PDDA) dispersion with an aqueous solution of EryB. PU alone was set as the control sample, and PU/Sap/PDDA was the sample without functionalized EryB. The antimicrobial efficacy of PU/Sap/PDDA/EryB nanocomposites was tested on 24-h biofilms of two *agr*-positive (S66, S68) and one *agr*-negative MRSA isolate. Figure 4 shows the effectiveness on biofilms formed on the modified nanomaterial compared to those grown on unmodified PU. The results showed that the non-irradiated samples did not manifest any antimicrobial efficacy. A significant reduction in biofilm cells was only observed after 10 min of irradiation with a green laser. The clinical isolates S66, S68, and S73 showed more than 100-fold reduction in colony-forming units (CFU mL^−1^) compared to the control biofilms formed by respective strains on PU alone. The effectiveness on the standard strain CCM 3953 was already tested and published in the work of Bugyna et al. [53] under the same conditions as in the present research. The inhibition was almost 1000-fold in the irradiated sample compared to the control biofilm grown on PU alone.

### 2.3. Analysis of the Effect of Photoactive Nanomaterial on hld Gene Expression Changes

**Figure 5:**
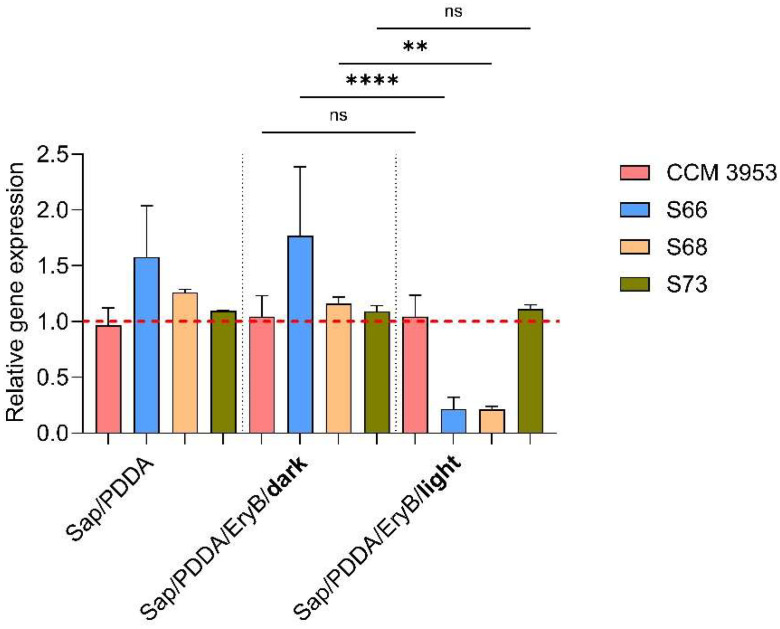
Relative expression of the *hld* gene on nanomaterial in 24-h biofilms before and after irradiation. The red line represents the control *S. aureus* biofilm for each strain formed on PU alone (set as 1); the *rpoD* gene was used as a housekeeping gene. Control sample with EryB but cultivated in the dark is labeled **dark**, and sample irradiated with a green laser for 10 min is labeled **light.** A *p* > 0.05 (ns) was considered non-significant; *p* < 0.01 (**) was very significant; and *p* < 0.0001 (****) was extremely significant.

The expression of the *hld* gene, which is located within RNAIII, was analyzed by qPCR. The results are summarized in Figure 5. Strain S66 slightly increased the expression of the *hld* gene in biofilms formed on both Sap/PDDA and non-irradiated composite with EryB, but this change was not significant. On the other hand, the results showed a significant decrease in the relative expression of the *hld* gene for the *agr*-positive strains (S66 and S68) after irradiation compared to the non-irradiated sample. The standard strains CCM 3953 and S73 showed no significant changes on the modified photoactive material compared to the non-irradiated samples.

## 3. Discussion

The Gram-positive bacterium *S. aureus* is a pathogen that can cause a broad spectrum of infections, ranging from minor skin lesions to severe, potentially fatal disease due to numerous virulence factors [1,2,3,4,5,6,7,8,57,58,59]. An important role in the regulation of these virulence factors is played by the *agr* QS system, which controls the expression of many genes via the effector molecule RNAIII [37,60,61]. The functionality of the *agr* QS system can be assessed using the CAMP test, which is based on the interaction of *S. aureus* hemolysins: the ability of β-hemolysin to inhibit α-hemolysin while increasing the activity of δ-hemolysin [62].

In our previous study, we presented an advanced nanocomposite modified with EryB that exhibited anti-biofilm properties [53]. Its effectiveness was tested on the standard strain *S. aureus* CCM 3953 by comparing two light sources (laser vs. LED light) and optimizing the irradiation time. The results showed an almost 1000-fold inhibition after 10 min of irradiation with a green laser. In addition, the formation of ROS was detected with 2,7-dichlorodihydrofluorescein diacetate. Garapati et al. [63] investigated EryB-based nanoparticles, which also exhibited anti-biofilm activity against *S. aureus* cells [63].

Based on our previous findings, the EryB-modified nanomaterial was tested using three MRSA isolates in this study. Since the formation of strong or weak biofilm is related to the *agr* system [13,17,64], the tested strains were also selected based on their activity of the mentioned QS system; they were representatives with high, lower, and no activity (CAMP test). It is also important to mention that the activity of the *agr* system is associated with effective eradication of internal and external ROS [15,65]. Since the antimicrobial effect of PDI is based on the production of large amounts of singlet oxygen and ROS, it was interesting to investigate the effect of PDI on the *agr* system. Its activity was determined by the expression of the *hld* gene, which is associated with RNAIII. Kumar et al. [65] confirmed that ROS are more accumulated in the *agr*-mutant compared to the wild-type. They also demonstrated that pre-treatment of *S. aureus* with menadione protected the mutant strain from the killing effect by H_2_O_2_, confirming that the *agr* system participates in the control of endogenous, but also exogenous ROS [65]. According to our results, no difference was observed between *agr*-positive and *agr*-negative strains in the response to PDI, as the irradiation of nanocomposites reduced the survival of biofilm cells of all strains by about 100-fold compared to the control biofilm grown on PU alone. Results from the qPCR showed a significantly decreased relative expression of the *hld* gene in the irradiated biofilm on the nanocomposite compared to the control sample on the PU alone and even to that formed on the non-irradiated nanocomposite, suggesting a significant reduction in the activity of the *agr* QS system. On the other hand, no change in the *hld* gene expression was observed in the *agr*-negative strains. Our results indicate that ROS generated during PDI probably contributed to the suppression of the *agr* system. This could be because of abolishing AgrA (the regulator of the *agr* system) though stress-mediated covalent binding of CoA (coenzyme A), leading to significantly decreased affinity towards the P2 and P3 promoters, which are critical for the functionality of the *agr* system (transcription of the RNAII and RNAIII) [66,67]. However, further targeted studies will be required to clarify how ROS specifically influence *agr* signaling pathways.

Several recent studies have demonstrated how PDI affects QS in different bacterial species. In *Pseudomonas aeruginosa*, PDI inhibited the LasIR system, which is responsible for QS, biofilm formation, and virulence [68]. Another study showed that indocyanine green-mediated PDI reduced the expression of the *abaI*, *agrA*, and *lasI* genes in mixed biofilms of *Acinetobacter baumannii, S. aureus*, and *P. aeruginosa* [69]. Some studies showed that sublethal doses of PDI with chlorine e6 and laser light (λ = 664 nm) induce stress responses of *S. aureus* and activate *agr*-dependent gene regulation. Mutant strains of *S. aureus* with a non-functional *agr* system were significantly more sensitive to PDI, suggesting that the *agr* system is involved in protection against oxidative stress induced by PDI [70,71]. Although clinical isolates of S66 and S68 showed significantly increased expression of the *hld* gene in planktonic cells, this expression was considerably reduced after irradiation of biofilms formed on photoactive nanocomposites. Our results show that the main antibacterial effect of PDI is mediated by ROS, as the survival of both *agr*-positive and *agr*-negative strains was reduced to a similar extent. At the same time, in *agr*-positive strains, PDI also reduced *hld* expression, indicating partial suppression of the *agr* system in addition to the general effect caused by ROS. Nevertheless, the nanocomposites with EryB displayed strong anti-biofilm effectiveness, suggesting that their activity is not dependent on the modulation of the QS pathway through the *hld* gene. These results suggest that photoactive nanocomposites can disrupt biofilms independently of QS regulation and, thereby, extend their potential applicability against MRSA clinical isolates.

## 4. Materials and Methods

### 4.1. Bacterial Strains and Microplate-Based Assessment of Biofilm Formation

The standard strain *S. aureus* CCM 3953 (Czech Collection of Microorganisms, Masaryk University, Brno, Czech Republic, corresponding to the strain RN4220 recommended for the CAMP test) and three clinical MRSA isolates, S66, S68, and S73 (isolated from hemocultures, Institute of Microbiology, Faculty of Medicine, Comenius University Bratislava, Slovakia), were used for experimental research. All strains were stored as stock cultures at −20 °C. Overnight cultures were incubated in Mueller–Hinton broth (MHB; Biolife, Milan, Italy) for 16 h at 37 °C. Samples were then transferred into fresh MHB and grown to an exponential phase OD_600_ = 0.5 (corresponding to ≈ 2 × 10^7^ cells mL^−1^) [12] at 37 °C with shaking at 150 rpm in an incubator (Thermo shaker PST-60H2-4 Biosan, Riga, Latvia). Columbia NutriSelect™ Plus blood agar (Becton Dickinson, Franklin Lakes, NJ, USA) was used to monitor the hemolytic activity of the *S. aureus* strains.

The 24-h biofilm was formed from the pre-incubated cultures (exponential phase). A 96-well plate was used to incubate the biofilm. A total of 100 µL from the pre-incubated culture and 100 µL of MHB were added to each well. The plates were incubated for 24 h at 37 °C. Biofilm was quantified by crystal violet staining [72,73]. Briefly, the biofilms were washed three times with 100 µL of phosphate-buffered saline (PBS; 137 mM NaCl, 2.7 mM KCl, 8 mM Na_2_HPO_4_, and 2 mM KH_2_PO_4_; CentralChem, Bratislava, Slovakia) and then stained with 0.1% crystal violet solution for 30 min at room temperature. Then the dye was extracted using 200 µL of 96% ethanol (CentralChem, Bratislava, Slovakia), followed by incubation for 30 min. Then 110 μL was transferred to a new plate. The absorbance was measured by a spectrophotometer (Dynex MRX-TC Revelation, Dynex Technologies, Chantilly, VI, USA) at OD_570_ nm to quantify the biofilms.

Biofilm samples of all strains were observed using fluorescence microscopy (Intraco Micro LM 600, Tachlovice, Czech Republic). The biofilms were previously grown on glass slides (12 mm diameter, Marienfeld-Superior, Lauda-Königshofen, Germany) in a 24-well plate. After 24 h of incubation, biofilms were washed with PBS, and then glass slides were transferred to a microscope slide, where a drop of fixation solution with DAPI (Fluoromount-G^TM^, with DAPI, Thermo Fisher Scientific, Waltham, MA, USA) had previously been applied. After 48 h of dark incubation at 4 °C, a drop of immersion oil (Immersol™ Immersion Oil, Zeiss, Germany) was applied to the slide, and biofilms were observed at a magnification of 1000× under the UV filter (excitation: 340–380 nm, emission: 435–485 nm).

### 4.2. Analysis of AGR System Functionality Using the CAMP Test

The CAMP test was performed on Columbia NutriSelect™ Plus blood agar supplemented with 5% sheep blood. Overnight cultures of all strains of *S. aureus* adjusted to ≈ OD_600_ = 0.5 were used for inoculation. The reference strain *S. aureus* CCM 3953 was streaked in the center of the agar plate. Clinical isolates of S66, S68, and S73 were inoculated perpendicular to the reference strain at a distance of 2–3 mm without touching. Plates were incubated statically for 24 h at 37 °C and then for another 24 h at 4 °C. The CAMP test was evaluated according to Traber et al. [36]. The functionality of the *agr* system was confirmed by enhanced hemolysis at the site of interaction of the tested and reference strains. The absence of enhanced hemolysis indicated a non-functional *agr* system.

### 4.3. Preparation of Nanocomposites and PDI

The nanocomposite was prepared as already described by Bugyna et al. [53]. Briefly, the nanomaterial was based on clay mineral, synthetic saponite (Sap; Kunimine Industries Co., Ltd., Tokyo, Japan), modified with poly(diallyldimethyl ammonium) cations (PDDA; Sigma Aldrich, Steinheim, Germany) and with or without EryB (Sigma Aldrich Corpo-ration, St. Louis, MO, USA) and was functionalized on a PU (PU; VARNISH-PU 2 KW, Iso-mat S.A., Thessaloniki, Greece) surface. Solutions of PDDA and EryB and a colloidal dispersion of Sap were prepared in deionized water and mixed. At first, the solution of PDDA was mixed with a colloidal dispersion of Sap (nPDDA/mSap = 1.5 mmol g^−1^), followed by the addition of EryB (nEryB/mSap = 1.5 mmol g^−1^) to the prepared colloidal dispersion of organically modified Sap. After being shaken at room temperature for 120 h, organoclay was filtered using membrane filters made from hydrophilic polytetrafluoro-ethylene (0.1 μm pore size, 47 mm diameter, Omnipore™, Millipore, Merck, Darmstadt, Germany). Then, a liquid PU was applied on the hybrid film with or without EryB. The nanocomposite was then left for 24 h in an air stream to dry. Then, it was peeled off the membrane. The nanocomposite disks PU/Sap/PDDA or PU/Sap/PDDA/EryB were cut into smaller pieces (1 cm^2^), sterilized by UV (10 min on both sides), and fixed with 2% agarose (150 µL) to the bottom of the wells of a 24-well plate (TC Plate, Sardstedt AG & Co., Ltd., Nümbrecht, Germany).

The prepared nanocomposite was characterized by attenuated total reflectance infrared (ATR IR) spectroscopy, absorption and fluorescence spectroscopy, and X-ray diffraction in our previous study [53]. Briefly, the absorption maximum of EryB adsorbed on the nanomaterial applied on PU matrix was at 542 nm, with a shoulder at 505 nm, which meant that a green laser with an emission wavelength of 532 nm was suitable for irradiation of the prepared samples, because its emission wavelength falls within the absorption spectrum of EryB. The emission maximum of EryB being adsorbed on the nanocomposite applied on the PU matrix was at 578 nm. The maximum adsorption capacity of EryB on Sap/PDDA nanoparticles was determined using absorption spectroscopy. It was found that the maximum adsorption capacity of EryB on Sap modified with PDDA polycation (1.5 mmol per 1 g of Sap) particles was 0.397 mmol·g^–1^. ATR IR spectroscopy was used to detect the presence of individual components and functional groups on the surface of the prepared samples. From the measured infrared spectra of pure components, as well as the nanomaterial, it could be seen that the surface of PU was covered with Sap/PDDA or Sap/PDDA/EryB (vibrational bands of pure PU disappeared, while new vibrational bands belonging to Sap, PDDA polycation, and EryB appeared in the infrared spectra).

A 24-h biofilms of CCM 3953 and clinical isolates were prepared in the 24-well microtiter plate by adding 0.5 mL of culture (OD_600_ = 0.5) and 0.5 mL of MHB to each well. After 24 h of static incubation at 37 °C, the medium was removed, and 50 µL of PBS was added to the wells to prevent dehydration during subsequent irradiation. The distance between the laser tip and the bottom of the 24-well plate was set to 5 cm during irradiation with a green laser (λ = 532 nm, 100 mW, Alligator, MZTech s.r.o., Košice, Slovakia) for 10 min. After irradiation, the samples were transferred to Eppendorf tubes with 1 mL PBS, sonicated (Branson 200 ultrasonic cleaner, Danbury, CT, USA) for 5 min, vortexed (Vortex V-1 plus, Biosan, Riga, Latvia) for 5 min, serially diluted, and inoculated on MHA. After 24-h incubation at 37 °C, (CFU mL^−1^) were calculated.

### 4.4. Isolation of RNA and qPCR

Planktonic cells in the exponential phase and 24-h biofilms grown on nanocomposites were used for RNA isolation. Planktonic cells were pre-incubated to OD_600_ = 0.5 and centrifuged (Universal 32 R, Hettich Zentrifugen, Tuttlingen, Germany) for 5 min at 2152× *g*. The biofilms grown on nanocomposite were irradiated with a green laser for 10 min (according to the protocol described in Section 4.3). After irradiation, biofilms were centrifuged for 5 min at 2152× *g*. Then, 150 μL of PBS and 20 μL of lysozyme (Fluka Analytical, Taufkirchen, Germany, 20 mg/mL) were added to the pellet, and the samples were vortexed and incubated for 30 min at 37 °C. Subsequently, 200 μL of Homogenization Solution, 200 μL of Lysis Buffer, and 200 μL of Lytic Enhancer (all provided by Maxwell RSC miRNA Tissue Kit, Promega, Madison, WI, USA) were added. Sterile glass beads (0.5 µm and 0.1 µm, 1:1 ratio, Thermo Fisher Scientific, Waltham, MA, USA) were added to the tubes, and the samples were vortexed for 20 min at 3500 RPM and centrifuged for 5 min at 10,413× *g*. Then 30 μL of proteinase K (provided by the Maxwell RSC miRNA Tissue Kit, Promega, Madison, WI, USA) was added to the tubes, and the samples were incubated for 15 min at 37 °C in a thermoblock (Termoblock MD-01N-110/220, Major Science, Saratoga, CA, USA) and centrifuged (5 min, 10,413× *g*).

For RNA isolation, a Maxwell RSC instrument (Promega, Madison, WI, USA) was used. A total of 600 µL of each sample, 10 µL of DNase (the Maxwell RSC miRNA Tissue Kit, Promega, Madison, WI, USA), and 50 µL of Nuclease-free water (Thermo Fisher Scientific, Waltham, MA, USA) were added to the cartridge. After isolation, samples were kept on ice. RNA concentration and purity was determined using a NanoDrop Spectrophotometer ND-1000 (NanoDrop Technologies, Wilmington, DE, USA), and samples were stored at −80 °C until used. Then, RNA samples were reverse-transcribed into cDNA using the Maxima First Strand cDNA Synthesis kit for RT-qPCR (Thermo Fisher Scientific, Waltham, MA, USA) according to the manufacturer’s instructions; cDNA samples were stored at −20 °C until used.

The qPCR was used to analyze the expression of the *hld* gene (forward: 5′-GAAGGAGTGATTTCAATGGCACAAG-3′; reverse: 5′ GAAAGTAATTAATTATTCATCTTATTTTTTAGTGAATTTG-3′) [37], normalized to the housekeeping gene *rpoD* (forward: 5′-CAC GAG TGA TTG CTT GTC-3; reverse: 5′-GAT ACG TAG GTC GTG GTA TG-3′) [74]. All primers were synthesized by Metabion International AG Planegg/Steinkirchen, Germany. Separate qPCR mastermixes were prepared for the *hld* gene and the housekeeping gene *rpoD*. The qPCR thermal protocol was performed as recommended by the manufacturer. HOT FIREPol^®^ EvaGreen^®^ qPCR Mix Plus (ROX) 5x (Solis BioDyne OÜ, Tartu, Estonia) and LightCycler^®^ PRO System (Roche Diagnostics, Indianapolis, IN, USA) were used for qPCR. The annealing temperature was set to 55 °C for 20 s. Data were analyzed in LightCycler PRO^®^ Development Software 1.0.0 (Roche Diagnostics, Indianapolis, IN, USA). Relative gene expression was calculated using the 2^ΔΔ^CT method [75], with *rpoD* as the reference gene [74]. For the experiment with planktonic cells, the relative expression of the *hld* gene was normalized to *S. aureus* CCM 3953, which was set to a value of 1. For experiments with biofilms on nanocomposites, changes in the *hld* gene expression were calculated using biofilm on non-modified PU of each strain as a control sample set to 1.

### 4.5. Statistical Analysis

Statistical significance was determined with Student’s *t*-test using the GraphPad Prism software 10.6.0 (Graph Pad, San Diego, CA, USA). *p* < 0.05 (*) was considered statistically significant; *p* < 0.01 (**); *p* < 0.001 (***); *p* < 0.0001 (****). *p* > 0.05 (ns) was considered non-significant.

## 5. Conclusions

The presented work showed that the photoactive nanocomposite functionalized with EryB can modulate the *agr* QS system in MRSA isolates. Since biofilm formation did not significantly increase despite the decreased relative expression of the *hld* gene—the most important marker for RNAIII activity and functionality of the *agr* system—it is assumed that biofilm formation is affected by genes that are independent of this system. In addition, the nanocomposite functionalized with EryB significantly decreased the survival of biofilms formed by both *agr*-negative and *agr*-positive strains after irradiation.

The presented photoactive nanocomposite represents a promising direction for further research, considering the potential of modified polymers for various applications, particularly in healthcare manufacturing, where specific surface properties are essential to reduce microbial colonization.

## Data Availability

Data have been published to Zenodo: https://doi.org/10.5281/zenodo.17295144.
